# A Loss Separation-Based Dynamic Jiles–Atherton–Bingham Model for Magnetorheological Dampers

**DOI:** 10.3390/s26041259

**Published:** 2026-02-14

**Authors:** Ying-Qing Guo, Yu Zhu, Yang Yang

**Affiliations:** 1 College of Mechanical and Electronic Engineering, Nanjing Forestry University, Nanjing 210037, China; zhuyu115@njfu.edu.cn; 2 China-Pakistan Belt and Road Joint Laboratory on Smart Disaster Prevention of Major Infrastructures, Southeast University, Nanjing 210096, China; 101013479@seu.edu.cn

**Keywords:** magnetorheological damper, multi-mechanism loss, dynamic Jiles Atherton hysteresis model, particle swarm-genetic algorithm

## Abstract

Magnetorheological (MR) dampers exhibit significant hysteretic nonlinearities, particularly under dynamic operating conditions, where accurately modeling the complex coupling between magnetic flux density and excitation current remains challenging. To overcome the limitations of the conventional static Jiles–Atherton (JA) model in capturing dynamic hysteresis responses, a dynamic JA model incorporating multiple loss mechanisms (LS-DJAM) is proposed for MR dampers. Building on loss separation theory, the model integrates eddy current and excess loss mechanisms to more accurately represent the dynamic hysteresis behavior of MR dampers. By coupling the Bingham mechanical model, a magneto-mechanical constitutive relation for MR dampers is established. Furthermore, to enhance the accuracy of LS-DJAM parameter identification, a hybrid particle swarm optimization–genetic algorithm (PSO–GA) is developed. Genetic operators are embedded within the PSO framework to strengthen the global search capability and mitigate premature convergence, thereby enabling efficient LS-DJAM parameter identification. The proposed LS-DJAM, identified via the PSO–GA, significantly enhances the modeling of MR damper output forces. PSO–GA parameter estimation improves accuracy by over 60%, and the LS-DJAM reduces the maximum modeling error by 87.5% compared with the conventional JA model. It accurately captures the dynamic hysteresis characteristics of MR dampers, providing a robust theoretical basis and practical framework for high-performance control and engineering optimization.

## 1. Introduction

Magnetorheological (MR) dampers are semi-active controllable vibration damping devices that primarily exploit the rheological properties of MR fluids. MR fluids consist of ferromagnetic particles, base fluids, and additives [1], and their fundamental mechanism involves a controllable solid–liquid phase transition. Under an external magnetic field, the suspended magnetic particles polarize, aggregate, and align along the field direction, thereby forming stable chain-like structures. This alignment markedly increases the fluid’s yield stress, resulting in solid-like behavior. Once the external field is removed, the chain structure destabilizes and the fluid rapidly returns to its original flowing state [2]. At present, MR dampers are widely applied in diverse fields such as machinery, automotive engineering, and civil engineering [3,4,5,6].

In the field of MRD research, numerous domestic and international scholars have developed various models to characterize their complex mechanical behaviors, thereby achieving effective vibration control. For instance, Viadero-Monasterio [7] developed a robust static output feedback control strategy for semi-active suspensions, demonstrating that simplified damper models are sufficient to achieve effective vibration suppression within closed-loop systems. Among these, the Bingham model [8], Bouc–Wen model, Herschel–Bulkley model, Biviscous model, and the Sigmoid model [9,10] are widely recognized as representative formulations. The Bingham model is favored for its structural simplicity and clear physical interpretation of parameters, effectively capturing the yield characteristics and force–displacement hysteresis of MR dampers, and is thus extensively used in engineering analysis and control simulation. The Bouc–Wen model, with its strong nonlinear fitting capability, has been widely applied to dynamic characterization and semi-active control studies. The Herschel–Bulkley and Biviscous models, offering improved accuracy in viscoelastic fluid modeling, are suitable for analyzing damping responses under varying shear rates. Building on these foundations, several studies have further addressed model uncertainty and dynamic response characteristics. Chen [11] proposed a stochastic Bingham model for MR dampers by treating certain input variables as random variables via the random factor method, and subsequently extended it to a random Bingham model through an algebraic synthesis approach, while Li [12] employed a modified smoothed particle hydrodynamics (SPH) method, incorporating periodic density reinitialization and artificial stress, to perform numerical investigations of the Bingham model for fluid flow. Yang [13] proposed a modified Bouc–Wen model, which significantly improved the fitting accuracy of MR damper dynamic hysteresis behavior. The inherent hysteretic nonlinearity of MR dampers poses significant challenges for accurate modeling and control. The existing mechanical models, such as the Bingham and sigmoid models, either oversimplify hysteresis or lack a physical basis for it, limiting their ability to predict damping forces accurately. Moreover, most models focus on mechanical behavior while neglecting the magnetic hysteresis within the damper core, whose coupling with the mechanical response can lead to substantial errors under dynamic magnetization. Therefore, a comprehensive mechanical model that accurately captures the nonlinear magneto-mechanical hysteresis is essential for the precise prediction and control of MR damper performance.

Hysteresis in ferromagnetic materials has been extensively studied. The Preisach model (1935) represents magnetic materials as ensembles of dipoles governed by hysteresis operators, with total magnetization expressed as their superposition. Earlier, Prandtl had introduced a framework for modeling plasticity, which Ishlinskii extended in the 1940s by proposing a mathematical formulation for mechanical hysteresis. The resulting Prandtl–Ishlinskii model effectively simulates diverse hysteresis phenomena through the superposition of linear systems [14] and is valued for its simplicity, few parameters, and analytical inverse [15]. Vatandoost et al. [16] proposed a Reduced Prandtl–Ishlinskii model by incorporating a stop-operator into the traditional PI framework. This modification reduces the number of parameters to only seven, significantly lowering computational costs while maintaining modeling accuracy. The Stoner–Wohlfarth model (1948) provided a vector framework to simulate magnetization processes [17,18].The Bouc–Wen model (1967, refined in 1976) was initially developed to describe material hysteresis and is now widely applied to nonlinear mechanical systems, particularly under large magnetic field variations [19,20]. The Jiles–Atherton (JA) model (1986) is a domain wall-based differential formulation derived from energy conservation principles [21]. The model is derived based on the principle of energy conservation during the magnetization of soft magnetic materials [22] and has gained popularity due to its convenient parameter identification and computational efficiency [23,24].

Although the Jiles–Atherton (JA) model effectively captures the physical characteristics of ferromagnetic materials, its reliance on differential equations and five parameters restricts its engineering applicability. Accurate identification of these parameters is therefore crucial. Leite et al. [25] applied genetic algorithms to identify JA parameters, achieving high accuracy but still facing the issue of local optima. Marion [26] employed multi-objective particle swarm optimization (PSO), yet the inability to escape local optima remained. Guo et al. [27] proposed an adaptive weighted PSO, which enhanced population diversity and avoided premature convergence, improving parameter identification and prediction accuracy. Li [28] introduced an SA–PSO algorithm, further accelerating convergence compared to conventional PSO. Neural network-based approaches have also been explored. Trapanese [29] trained networks on known hysteresis loops, while Wang [30] used neural networks to identify key JA parameters with high accuracy. However, both approaches suffer from strong data dependence. Overall, optimization algorithms reduce data reliance but are limited by computational efficiency, whereas neural networks achieve higher accuracy at the cost of dataset dependence.

The Jiles–Atherton (JA) model exhibits high computational complexity, limited physical interpretability, and restricted applicability (e.g., single-domain or quasi-static conditions), making it challenging to implement in MR dampers. Therefore, this study adopts the dynamic Jiles–Atherton model as the primary framework. The conventional dynamic Jiles–Atherton model, based on the empirical method of loss separation (EMLS), offers convenience and high computational efficiency [31,32]. However, the EMLS relies on empirical data fitting, lacks generality, and exhibits limited predictive accuracy under complex magnetization conditions or high-frequency dynamic behavior. In this study, a dynamic Jiles–Atherton hysteresis model based on loss statistical theory (LS-DJAM) is employed, which computes static hysteresis, eddy current, and excess losses separately using the static hysteresis model and loss statistical theory. Under dynamic magnetic fields, the energy loss of the material is no longer a purely static process. LS-DJAM accounts for the influence of frequency and magnetic field intensity on energy loss, providing a more accurate representation of the MR damper hysteresis during operation. Furthermore, when coupled with the Bingham model, it establishes a magneto-mechanical constitutive relationship [33], enabling simultaneous description of both the hysteretic effects and plastic flow characteristics under applied magnetic fields, thereby offering a comprehensive depiction of the nonlinear mechanical behavior of MR dampers. To enhance the model’s practicality and predictive accuracy, a hybrid particle swarm–genetic algorithm (PSO–GA) is employed to identify the key parameters of the LS-DJAM. Experimental results demonstrate that the model outputs closely match the measured mechanical responses, validating the effectiveness and superiority of the proposed method in complex nonlinear modeling and control.

## 2. Mechanical Model of MR Dampers

### 2.1. Bingham Model

**Figure 1:**
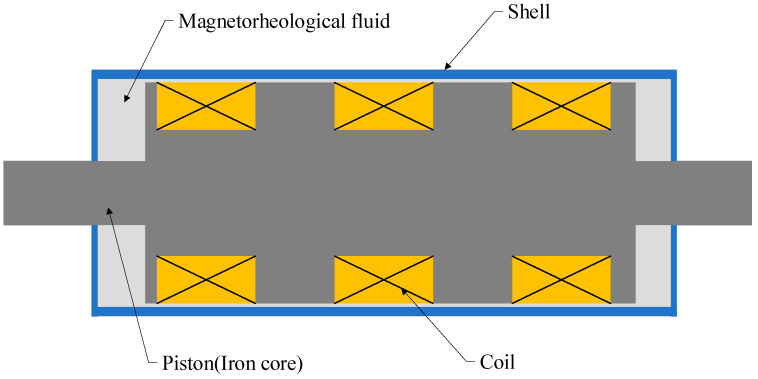
Schematic diagram of a shear–valve-type MR damper structure.

MR dampers operate in three modes: shear mode, valve mode, and shear–valve mode. This study focuses on a double-rod shear–valve MR damper, whose structural configuration is illustrated in Figure 1. Compared to the pure shear mode (which generates relatively low forces) or the pure valve mode, the shear–valve mode effectively combines the pressure drop induced by fluid flow with the shear stress generated by the piston motion. This combination significantly maximizes the damping force density and energy dissipation efficiency, making it the most suitable configuration for large-scale civil engineering applications where high damping forces and compact device sizes are essential. The device primarily consists of MR fluid, a stainless-steel shell, a piston (iron core), and an excitation coil. The hysteresis phenomenon mainly originates from ferromagnetic materials (e.g., DT4E pure iron) in the core and piston, which together account for more than 40% of the total damper volume. However, the influence of MR fluid on the hysteresis characteristics of MR dampers is negligible and can thus be disregarded [34]. Consequently, a pronounced nonlinear hysteretic relationship arises between magnetic induction intensity and the applied magnetic field, which constrains the effectiveness of MR dampers in applications demanding precise vibration control and intelligent structural systems.

**Figure 2:**
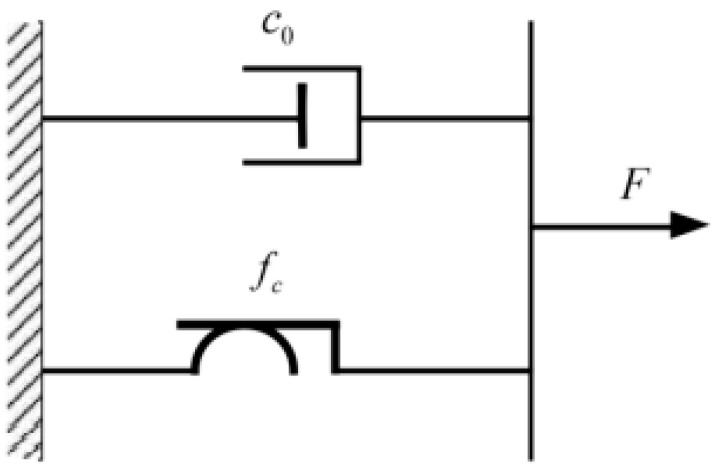
Bingham mechanical model.

The Bingham model is employed to characterize the mechanical behavior of the MR damper, consisting of a Coulomb friction element in parallel with a linear viscous element, as illustrated in Figure 2.

The stress–strain relationship of the double-rod shear–valve MR damper is expressed as follows:

(1) $$\tau =\tau_{0}\mathrm{sgn}(\dot{\gamma })+\eta \dot{\gamma }$$

The shear stress τ is composed of two components: $\tau_{0}$ is the yield stress caused by applied magnetic field, and the other is the viscous damping force, which is related to the dynamic viscosity coefficient η and the shear strain rate of MR fluid $\dot{\gamma }$.

In a shear–valve MR damper, the damping force can be decomposed into shear-mode and valve-mode components. During operation, the reciprocating motion of the piston drives the MR fluid to flow through the cavities and gaps on both sides. This flow can be regarded as a combination of shear flow and differential-pressure flow. Shear flow is primarily governed by the shear properties and velocity of the MR fluid, whereas differential-pressure flow is determined by the channel geometry and piston length. In the shear–valve mode, the shear flow of the MR fluid produces a damping force given by

(2) $$F=\left(\frac{12\eta LA_{P}^{2}}{\pi Dh^{3}}+\frac{L\pi D\eta }{h}\right)v+(\frac{3A_{P}L}{h}\left.+L\pi D\right)\tau_{0}\mathrm{sgn}(v)$$

where *L* represents the effective length of the piston; *h* denotes the effective gap between the piston and the cylinder; *D* signifies the diameter of the piston; υ represents the relative motion speed of the piston and the cylinder; *A_p_* indicates the effective area of the piston, sgn(υ), which is instrumental in determining the direction of the pressure difference flow in the cylinder relative to the piston.

The double-rod MR damper possesses a relatively simple magnetic circuit and structural configuration, making it suitable for various vibration control applications. The MR fluid employed in this study was developed by Prof. Xu’s team. Incorporating the rheological parameters of this specific fluid, the resultant output force of the double-rod shear–valve MR damper can be derived as [35]

(3) $$\begin{array}{l} f_{d}=\frac{12\eta LA_{P}^{2}}{\pi Dh^{3}}v+\frac{3A_{P}L}{h}\mathrm{sgn}(v)\cdot \\ \left(146960B^{0.5811}V^{1.706}\rho^{0.2}Ms^{0.8}+1083.2V-287.03\right) \end{array}$$

The total damping force *f_d_* is decomposed into two components: the viscous damping force (the first term), which is governed by the base fluid viscosity, and the field-dependent Coulomb damping force (the second term).

In this expression, *v* denotes the piston velocity, and η represents the zero-field viscosity of the MR fluid. The geometric parameters *L*, *D*, *h*, and *A_p_* correspond to the effective length of the annular duct, the mean diameter, the gap thickness, and the effective piston area, respectively.

This empirical regression model quantifies the nonlinear contributions of the magnetic flux density *B*, the particle volume fraction *V*, the fluid density ρ, and the saturation magnetization *M_s_*. The numerical coefficients are fitting parameters derived from experimental characterization, reflecting the sensitivity of the yield stress to each physical variable.

The output force of an MR damper is generally controlled by adjusting the input current, which in turn modifies the internal magnetic field. However, magnetic hysteresis introduces significant nonlinearity into the relationship between magnetic flux density *B* and current *I*; the relationship between *B* and *I* is typically influenced by the coil parameters of the damper and the external magnetic field magnetization *M*, thereby affecting the accuracy of the output force. Therefore, it is essential to establish a hysteresis model that can accurately describe the *B–I–M* relationship.

### 2.2. Jiles–Atherton Hysteresis Model

The hysteresis characteristics of the piston and iron core in an MR damper play a critical role in determining the accuracy of its output under varying magnetic fields. By contrast, the contribution of the MR fluid to the overall hysteresis behavior of the MR damper is negligible and can thus be ignored. Therefore, accurate modeling of the hysteresis properties of the piston and iron core is essential for improving MR damper performance.

The JA hysteresis model can be obtained [36]:

(4) $$\frac{dM}{dH}=(1-c)\frac{Man-Mirr}{k\delta -\alpha (Man-Mirr)}+c\frac{dMan}{dH}$$

where *k* is the hysteresis loss parameter; α represents the molecular field coefficient; *c* represents reversible susceptibility; and *δ* is a parameter indicating the direction of change in the magnetic field; when d*H*/d*t* > 0, the magnetic field gradually increases with time, and *δ* = 1; and when d*H*/d*t* < 0, the magnetic field gradually decreases with time, and *δ* = −1.

The JA model characterizes the interaction between magnetization *M* and magnetic field *H*. To analyze MR damper behavior comprehensively, the relationship between magnetic flux density *B* and magnetic field strength *H* must be established. Magnetic flux density *B* is related to magnetization *B* and magnetic field *H* by

(5) $$B=\mu_{0}\left(H+M\right)$$

Direct measurement of *H* is often challenging, whereas the excitation current *I*, closely related to *H*, is readily measurable:

(6) $$H=\frac{N\cdot I}{L_{e}}$$

where $\mu_{0}$ is the vacuum permeability, *N* represents the number of coil turns, *I* is the excitation current, and *L_e_* is the effective magnetic circuit length.

Considering the dynamic nature of the JA model, differentiating Equation (4) with respect to time *t* yields a time-dependent differential equation:

(7) $$\frac{dM}{dt}=(1-c)\frac{Man-Mirr}{k\delta -\alpha (Man-Mirr)}\frac{dH}{dt}+c\frac{dMan}{dt}$$

### 2.3. Dynamic Jiles–Atherton Model Based on Loss Statistics Theory

The traditional static JA model suffers from low accuracy, whereas the dynamic JA model based on conventional empirical formulas lacks clear physical meaning and performs poorly in estimating dynamic iron losses. Therefore, an improved dynamic JA model (LS-DJAM) is necessary. Bertotti [37] assumes a uniform distribution of magnetic flux density within ferromagnetic materials. Based on domain theory and the statistical distribution of magnetic domains, Bertotti derived analytical expressions for the eddy current loss *W_eddy_* and residual loss *W_exc_*.

(8) $$W_{eddy}=k_{e}{\int_{0}^{T}\left(\frac{dB}{dt}\right)}^{2}dt=\frac{\sigma d^{2}}{12}{\int_{0}^{T}\left(\frac{dB}{dt}\right)}^{2}dt$$

(9) $$W_{exc}=k_{a}{\int_{0}^{T}\left(\frac{dB}{dt}\right)}^{3/2}dt=\sqrt{\sigma SGV_{0}}{\int_{0}^{T}\left(\frac{dB}{dt}\right)}^{3/2}dt$$

where *k_e_* represents the eddy current effect intensity, and where $k_{e}=\sigma d^{2}/12$ with *d* being the thickness of the ferromagnetic object and σ the resistivity of the ferromagnetic material; *k_a_* = $\sqrt{\sigma SGV_{0}}$, where *S* denotes the cross-sectional area of the material. *G* is a dimensionless constant, *G* = 0.1357, and *V*_0_ is a statistical parameter characterizing the local magnetic field distribution of the magnet.

The magnetic field component associated with the eddy current loss is given by

(10) $$\frac{dW_{eddy}}{dt}\frac{\sigma d^{2}}{12}{\left(\frac{dB}{dt}\right)}^{2}$$

(11) $$H_{eddy}\left(t+\Delta t\right)=\frac{\sigma d^{2}}{12}\frac{\Delta B}{\Delta t}$$

During magnetization, changes in the domain walls result in residual loss, and the unit residual loss, based on loss statistics theory, can be expressed as

(12) $$\frac{dW_{exc}}{dt}=\sqrt{\sigma SGV_{0}}{\left(\frac{dB}{dt}\right)}^{3/2}$$

The magnetic field component corresponding to the residual loss is given by

(13) $$H_{exc}\left(t+\Delta t\right)=\sqrt{\sigma SGV_{0}}{\left|\frac{\Delta B}{\Delta t}\right|}^{1/2}$$

By integrating the dynamic field separation method with statistical loss theory, the LS-DJAM model is obtained:

(14) $$\begin{array}{l} \frac{dM}{dB}=\frac{\delta_{M}\left(M-M_{an}\right)-k\delta c\frac{dM_{an}}{dH_{e}}}{\mu_{0}\left\{\left(1-\alpha \right)\left[\delta_{M}(M-M_{an})-k\delta c\frac{dM_{an}}{dH_{e}}\right]\right.\left.-k\delta \right\}}+\\ \frac{\frac{\sigma d^{2}}{12}\left|\frac{\Delta B}{\Delta t}\right|+\sqrt{\sigma SGV_{0}}{\left|\frac{\Delta B}{\Delta t}\right|}^{1/2}}{\mu_{0}\left\{\left(1-\alpha \right)\left(M-M_{an}-k\delta c\frac{dM_{an}}{dH_{e}}\right)\right.\left.-k\delta \right\}} \end{array}$$

The eddy current loss *W_eddy_* expression (10) involves only the solution for the derivative of magnetic flux density *B* with respect to time *t*. Therefore, the numerical value of *W_eddy_* can be directly determined from the eddy current loss expression, taking the input *B = B_m_cos*(2*πft*).

The expression for eddy current losses in loss statistics theory is

(15) $$W_{eddy}=\frac{{\left(\pi d\right)}^{2}\sigma }{6}{B_{m}}^{2}f$$

The residual loss expression is derived as follows:

(16) $$W_{exc}=8.76\sqrt{\sigma SGV_{0}}{B_{m}}^{1.5}f^{0.5}$$

In Equation (16), the statistical parameter *V*_0_ depends on the peak alternating magnetic flux density *B_m_*, making direct calculation challenging. Based on the relationship between total loss *W_total_*, hysteresis loss *W_hys_*, eddy current loss *W_eddy_*, and residual loss *W_exc_*, the difference between total loss and eddy current loss *W_total_* – *W_eddy_* exhibits a linear relationship with the square root of frequency *f* ^0.5^. The intersection point of the corresponding function with the vertical axis represents the hysteresis loss component *W_hys_*, with a slope *k_v_* = $8.76\sqrt{\sigma SGV_{0}}$. Once the slope *k_v_* is determined, the statistical parameter *V_0_* for residual loss can be efficiently computed.

## 3. Parameter Identification of LS-DJAM

Equation (14) reveals that the fidelity of the LS-DJAM magnetic hysteresis loop is primarily controlled by five essential parameters (*k*, *β*, *α*, *c*, and *M_s_*), and their identification accuracy plays a decisive role in determining the overall modeling precision. To accurately capture the hysteresis behavior of the MR damper, these five static parameters must be identified. In this study, the PSO–GA is used for parameter identification.

### 3.1. Particle Swarm Optimization Algorithm

Particle swarm optimization (PSO) is inspired by the collective foraging behavior of bird flocks. Particles iteratively update their velocity and position based on an objective function that evaluates fitness, determining both individual and global best positions. The swarm adjusts accordingly to converge toward the global optimum. The velocity and position of particle *i* in the *j*th dimension are updated as

(17) $$\upsilon_{ij}(d+1)=\omega \upsilon_{ij}(d)+c_{1}r_{1}\left[{p_{ij}}^{best}(d)-xij(d)\right]+c_{2}r_{2}\left[{p_{gj}}^{best}(d)-x_{ij}(d)\right]$$

(18) $$x_{ij}(d+1)=x_{ij}(d)+\upsilon_{ij}(d+1)$$

where *d* denotes the current iteration count; *c*_1_ and *c*_2_ represent acceleration factors; *ω* is the inertia factor; *v_ij_* (*d*) indicates the *j*th component of particle *i* velocity vector at iteration *d*; *x_ij_* (*d*) denotes the *j*th component of particle *i* velocity vector at iteration *d*; *p_ij_^best^*(*d*) denotes the historically optimal solution found by particle *i* up to iteration *d*; *p_gj_^best^*(*d*) represents the globally optimal solution discovered by the entire particle swarm.

PSO seeks the global optimum based on the current best solution. It is simple, easy to implement, and offers high accuracy and rapid convergence, making it widely used. However, rapid convergence can reduce particle diversity, causing the algorithm to be trapped in local optima. Thus, improvements are needed to enhance the parameter identification accuracy.

### 3.2. PSO–GA

**Figure 3:**
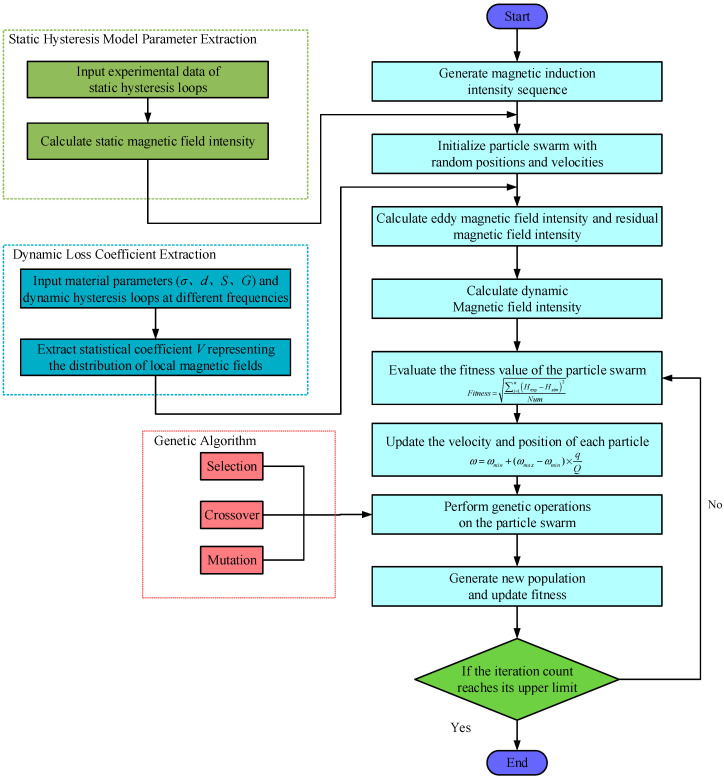
Flowchart of parameter identification of PSO–GA.

Incorporating genetic algorithm (GA) operations—selection, crossover, and mutation—into particle swarm optimization (PSO) markedly enhances information exchange and particle diversity, thereby improving global search capability and overall optimization performance. For particles *i* and *j*, the crossover operation on their positions and velocities can be described as follows:

(19) $$\left\{\begin{array}{l} x_{im}^{new}=x_{im}(1-\alpha )+x_{jm}\alpha \\ x_{jm}^{new}=x_{jm}(1-\alpha )+x_{im}\alpha \end{array}\right.$$

(20) $$\left\{\begin{array}{l} v_{im}^{new}=v_{im}(1-\beta )+v_{jm}\beta \\ v_{jm}^{new}=v_{jm}(1-\beta )+v_{im}\beta \end{array}\right.$$

where *α* and *β* are random numbers in the interval [0, 1]; *m* is a randomly chosen crossover dimension. After performing crossover to update the particle velocity and position, mutation is applied to produce new individuals:

(21) $$x_{im}^{new}=\left\{\begin{array}{l} x_{im}+(x_{max}-x_{im})\gamma (d),r\geq 0.5\\ x_{im}+(x_{max}-x_{im})\gamma (d),r\leq 0.5 \end{array}\right.$$

where *x_max_* denotes the upper bound of dimension *m*; *x_min_* denotes the lower bound of dimension *m*; $\gamma \left(d\right)=r_{3}{\left(1-t/T_{\max}\right)}^{2}$ represents the adaptive mutation step size; *r*_3_ is a random number in the interval [0, 1]; *d* is the current iteration count; *T_max_* denotes the maximum iteration count; *r* performs mutation operations by triggering random numbers within the interval [0, 1]. The algorithm flowchart is shown in Figure 3.

The algorithm steps are as follows:

(a) Generate the initial population. Initialize relevant parameters. Set the population size to 30 and the maximum iteration count to 150. The inertia weight decreases linearly between 0.8 and 0.2 during iterations, with $c_{1}=c_{2}=2$. The search space for all five model parameters is defined as the region within ±100% of their true values.

(b) Fitness calculation: The fitness function quantifies convergence toward the global optimum. The LS-DJAM parameter identification problem is formulated as minimizing the objective function, defined as the error between simulated and experimental magnetic field strengths:

(22) $$Fitness=\sqrt{\frac{\sum_{i=1}^{n}{\left(H_{\exp}-H_{sim}\right)}^{2}}{Num}}$$

where *H_exp_* denotes the experimental value of magnetic field strength *H*; *H_sim_* denotes the calculated value of the magnetic field strength *H*; and *Num* denotes the number of experimental data points.

(c) The inertial weight *ω* is decremented according to the following formula to generate a new inertial weight, thereby updating the particle’s velocity and position.

(23) $$\omega =\omega_{min}+(\omega_{max}-\omega_{min})\times \frac{q}{Q}$$

where *ω_min_* is 0.2, *ω_max_* is 1, *Q* is the total number of iterations, and *q* is the current iteration number.

(d) Recalculate the fitness values for each particle, then update the current particle swarm’s individual optimal solution and global optimal solution once more. Determine whether the termination condition is met; if satisfied, terminate the iterative optimization process; if not satisfied, proceed to step (e).

(e) Update individual and global best positions and perform genetic operations on particles. Apply crossover to selected particles to generate offspring, then apply mutation. Merge the new GA individuals with those updated by PSO to form a complete population, then return to step (b). The hyperparameter settings of the proposed PSO–GA are shown in Table 1.

## 4. Numerical and Experimental Analysis

### 4.1. Numerical Simulation Analysis

**Figure 4:**
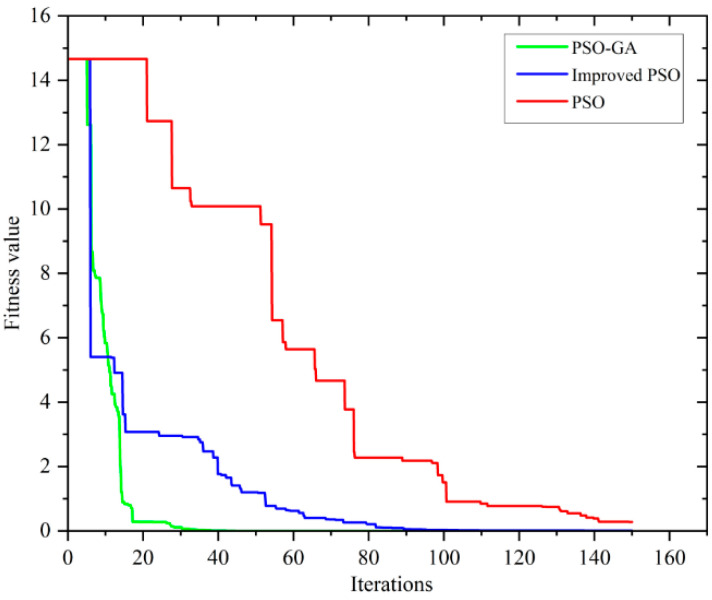
Diagram of the number of iterations of different algorithms under 50 Hz conditions.

**Figure 5:**
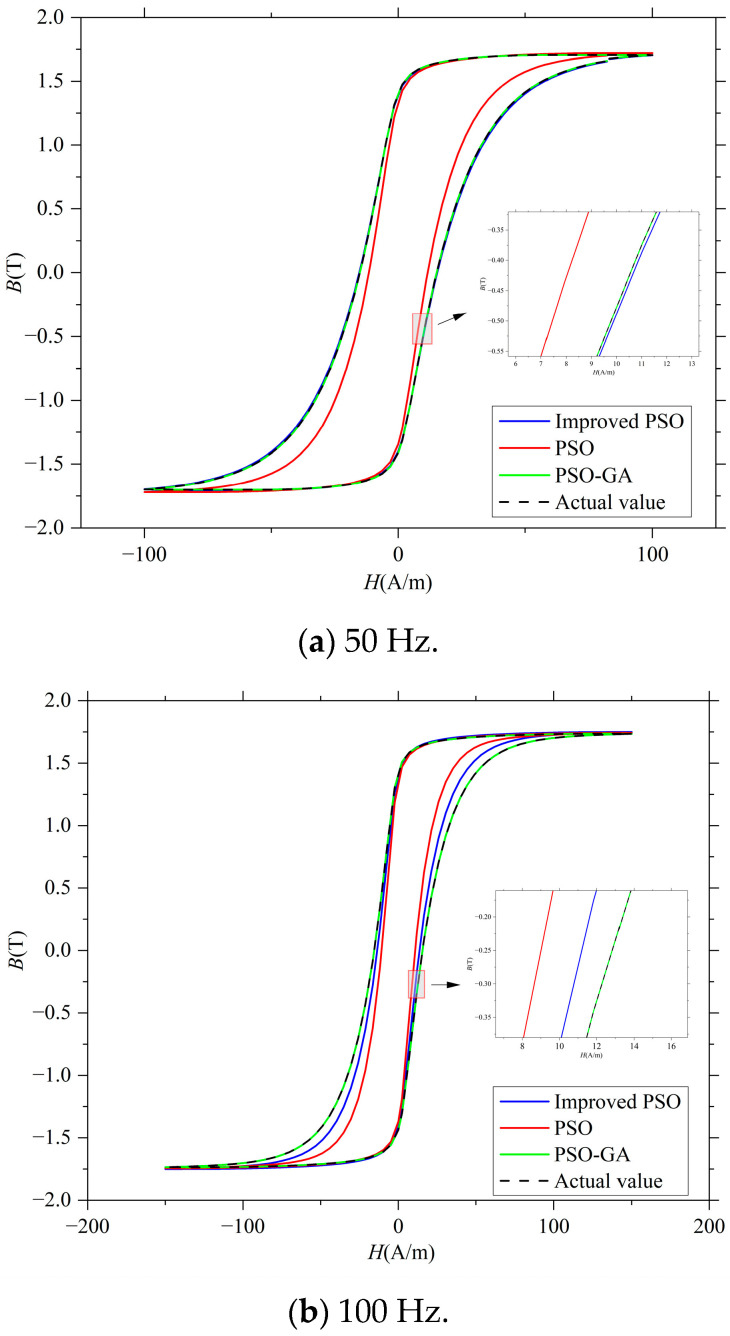

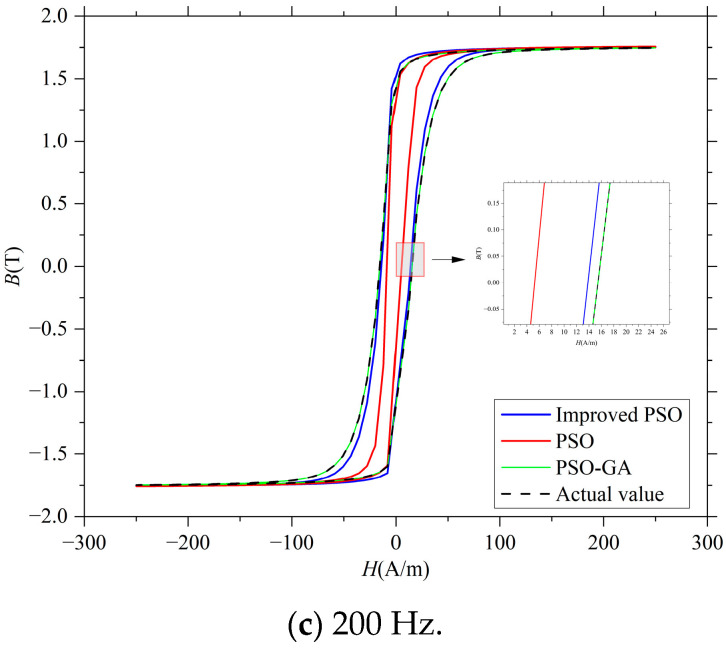
Analog hysteresis loops at different frequencies.

To validate the effectiveness of the proposed PSO–GA for LS-DJAM parameter identification, an LS-DJAM with known parameters from Reference [38] was selected as the fitting target. Using this *B–H* curve, the PSO–GA was employed to optimize the LS-DJAM parameters at different frequencies (50 Hz, 100 Hz, and 200 Hz), and the corresponding *B–H* curves were computed. The PSO–GA was initialized with a population size of 30 and a maximum iteration count of 150. The search space for all five model parameters was confined within ±100% of their true values. During iteration, the inertia weight *ω* decreased linearly from 0.8 to 0.2, while acceleration coefficients *c*_1_ and *c*_2_ were both set to 2. Additionally, for operating frequencies of 50 Hz, 100 Hz, and 200 Hz, the LS-DJAM hysteresis loops were optimized using both the standard PSO and the improved PSO algorithms. The hysteresis loops obtained by the three methods were compared. Parameter identification results are summarized in Table 2, while iteration histories and simulated hysteresis loops for each algorithm at different frequencies are presented in Figure 4 and Figure 5.

Table 1 summarizes the parameter identification results of the LS-DJAM using different optimization algorithms. The PSO–GA yields parameters closest to the theoretical values (*M_s_* = 0.1%, *α =* 0.4%, *a =* 0.08%, *c =* 0.2%, *k =* 0.3%), outperforming both the improved PSO (*M_s_* = 0.8%, α = 5.5%) and the standard PSO (*M_s_* = 2.3%, α =9.7%, k = 6.7%). These results confirm that PSO–GA provides the highest accuracy in *B–H* curve fitting.

As illustrated in Figure 4, PSO–GA exhibits the fastest and most stable convergence, reaching the global optimum within 30 iterations, while the improved PSO converges after approximately 90 iterations and the standard PSO remains trapped at a fitness value of 0.4 after 150 iterations. This demonstrates PSO–GA’s superior capability to escape local minima and ensure global convergence.

Figure 5 compares the simulated and measured hysteresis loops under different excitation frequencies. At 50 Hz, all algorithms achieve acceptable fits, but PSO–GA shows the lowest mean absolute error (MAE ≈ 0.02 T). As frequency increases to 100 Hz and 200 Hz, errors of the improved and standard PSO grow significantly (MAE = 0.08 T and 0.18 T, respectively), while PSO–GA maintains high precision (MAE ≤ 0.04 T). These findings highlight PSO–GA’s robustness and superior accuracy in dynamic hysteresis modeling across varying frequency conditions. Computational efficiency was evaluated over 20 independent trials. While the PSO–GA’s average runtime (125.4 s) exceeds the standard PSO (98.2 s) due to genetic operators, it remains faster than the improved PSO (167 s). This moderate cost is justified by substantial gains in the identification accuracy and global search capability. Crucially, PSO–GA is designed for offline identification. The resulting LS-DJAM relies on explicit algebraic equations with minimal computational load, enabling it to easily meet real-time control requirements (e.g., >1 kHz) on embedded systems.

**Figure 6:**
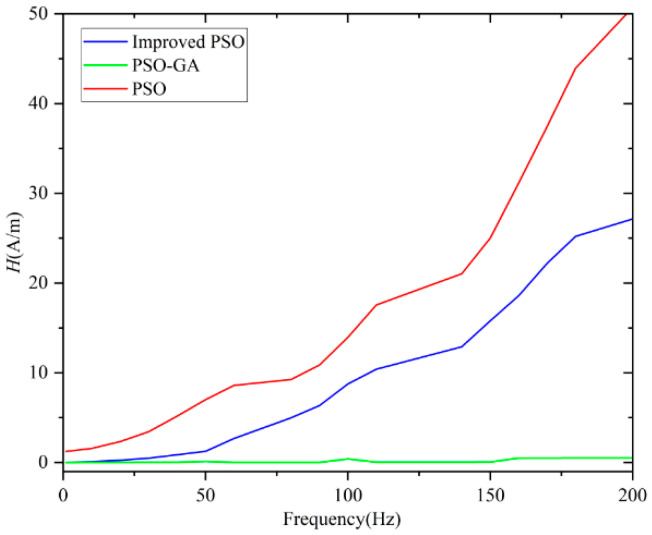
Algorithm error plot.

To further quantify the frequency-dependent performance, Figure 6 presents the variation in *B–H* curve fitting errors with excitation frequency, where the error between the simulated and experimental magnetic field intensities serves as the evaluation metric. The standard PSO exhibits a sharp error increase, indicating poor adaptability to high-frequency dynamics. The improved PSO shows moderate error growth, while PSO–GA maintains the smallest variation, highlighting its superior stability in dynamic modeling.

Overall, the LS-DJAM optimized by PSO–GA demonstrates higher parameter accuracy and faster convergence than the other algorithms. To further validate the advantages of incorporating multiple loss mechanisms, subsequent experiments compare the performance of LS-DJAM with the conventional JA model.

### 4.2. Simulation-Based Verification of the Magnetic Coupling Mode

**Figure 7:**
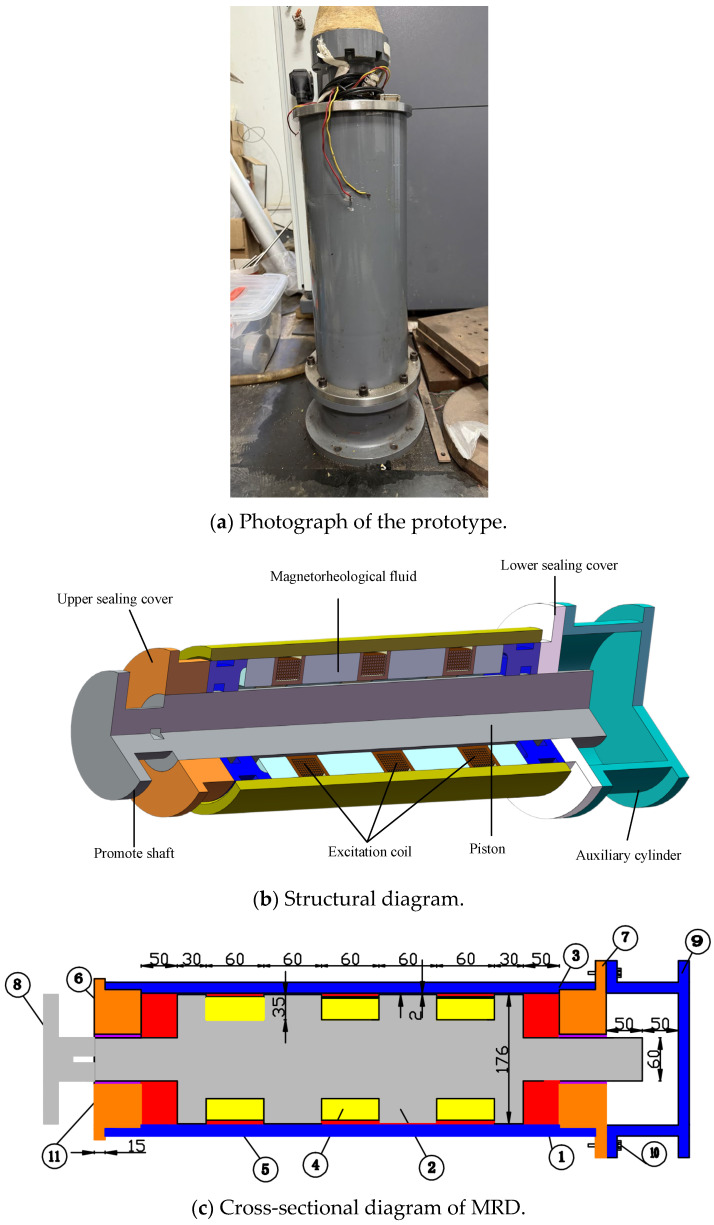
MRD-SEU-D050 magnetorheological damper.

This study employs a shear–valve-type MR damper developed by the Prof. Xu team at Southeast University [39], model MRD-SEU-D050. This damper is a three-coil MR damper with an integrated Hall sensor, manufactured based on theoretical and finite element analysis [40], as shown in Figure 7. The magnetorheological fluid used in this damper was also developed by the Prof. Xu team, with its performance parameters detailed in Table 3. The encapsulated magnetic particles employed in this damper feature low density and relatively high saturation magnetization, enhancing the magnetic rheological fluid’s resistance to sedimentation [41], as shown in Figure 7c, wherein 1. denotes the outer cylinder; 2. the piston; 3. the magnetorheological fluid (MRF); 4. the excitation coil; 5. the epoxy resin; 6. the upper sealing cover plate; 7. the lower sealing cover plate; 8. the propulsion shaft; 9. the auxiliary cylinder; 10. the bolts; and 11. the sealing rings. Each coil set consists of 1500 turns, with a total resistance of 14.3 Ω per set.

**Figure 8:**
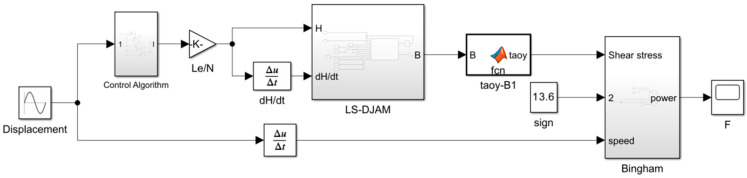
Bingham mechanical model of a magnetorheological damper.

To further assess the algorithm’s effectiveness in improving MR damper output damping force accuracy, a numerical simulation model of the MR damper was developed on the Simulink platform. The model integrates both the Bingham model and LS-DJAM, as illustrated in Figure 8, with the input port functioning as a real-time control interface. Notably, the model exhibits pronounced velocity-dependent behavior, requiring velocity time-series data obtained via numerical differentiation to establish the time-varying property correlations. The parameter module dynamically computes values based on the damper’s physical parameters, forming the core control system governing the magnetorheological effects.

Extraction of dynamic parameters for MR damper: Owing to the complex structure of the MR damper and the dominant hysteresis effect of the piston relative to other components, the extraction of dynamic parameters focuses solely on the piston. The piston has a diameter of *D =* 50 mm, yielding a cross-sectional area of *S =* π(*D*/2)^2^ *=* 1.96 × 10^−3^ m^2^. The piston is made of DT4-E electrical pure iron, with an electrical resistivity of *σ =* 1.0 × 10^−7^ Ω·m. Given that the outer diameter of the piston is 40 mm, the equivalent thickness *d* for the internal eddy current path is taken as 0.04 m. The statistical coefficient *V*_0_, which characterizes the local magnetic field distribution in the magnet, is generally determined from the linear relationship between the difference in total loss and eddy current loss (*W_total_* – *W_eddy_*) and the square root of frequency (*f*^0.5^):

(24) $$V_{0}=\frac{k^{2}}{{\left(8.76{B_{m}}^{3/2}\right)}^{2}\sigma SG}$$

**Figure 9:**
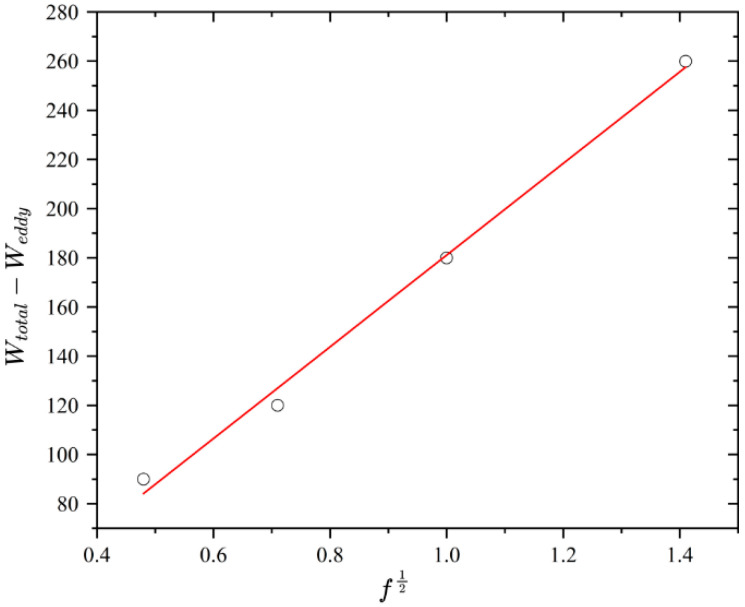
Linear relationship between $W_{total}-W_{eddy}$ and $f^{0.5}$.

Using experimental dynamic loss data at 0.1 Hz, 0.2 Hz, 0.5 Hz and 1 Hz, the fitting equation for *W_total_* – *W_eddy_* versus *f*^0.5^ is given by Equation (25). As shown in Figure 9, a linear relationship is observed, from which *V*_0_ is determined to be 0.0356.

(25) y=184.2x+5.87

**Figure 10:**
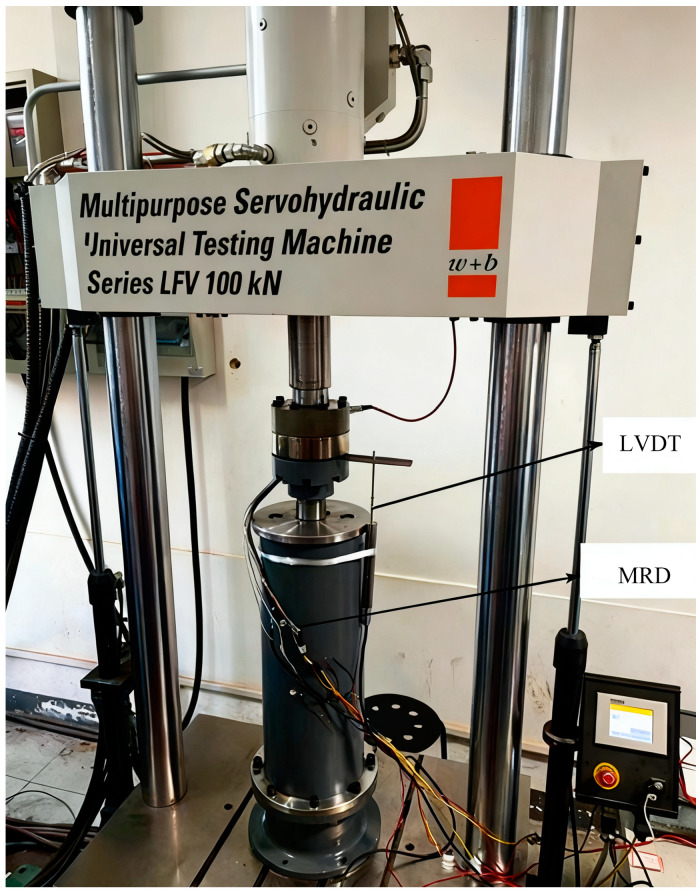
Experimental test system.

To ensure the accuracy and reliability of the damping force measurements, an MTS electro-hydraulic servo material testing system was employed as the excitation source. The experimental setup, depicted in Figure 10, comprises the following key subsystems:

(1) Mechanical Loading: A programmable MTS hydraulic servo-testing machine applied dynamic displacement excitations to the vertically mounted MRD. The system operated in displacement control mode with a precision of 0.01 mm.

(2) Power Supply: A custom-developed controller provided a continuous excitation current to the coils. The current was regulated from 0 A to 1.2 A, with stability monitored throughout the tests.

(3) Data Acquisition and Sensor Placement: Force and LVDT displacement signals were measured simultaneously by the MTS system’s built-in sensors. A high-precision load cell integrated into the actuator measured the instantaneous damping force, while the built-in LVDT monitored the relative displacement.

(4) Data Reliability: To verify the reproducibility of the experimental results, parallel experiments were conducted through multi-cycle loading. For each working condition, the damper was subjected to continuous excitation. Data from three independent stable cycles were extracted and averaged to eliminate transient effects and random noise. This averaged dataset was used for the subsequent model identification and validation.

During testing, the control current exhibited a nonlinear relationship with displacement, as defined by the correspondence criteria in Equation (26). Control strategies are critical for semi-active systems. In this study, a fuzzy controller was implemented to rapidly determine the damper’s control current, achieving superior control performance [42].

(26) $$I=\left\{\begin{array}{cc} 0 & \left|x\right|\leq 5\\ 0.2 & 5<\left|x\right|\leq 10\\ 0.4 & 10<\left|x\right|\leq 20\\ 0.6 & 20<\left|x\right|\leq 30\\ 0.8 & 30<\left|x\right|\leq 40\\ 1.2 & \left|x\right|>40 \end{array}\right.$$

To comprehensively evaluate the capability of the LS-DJAM in modeling rate-dependent hysteresis behavior, sinusoidal displacement excitations with frequencies of 0.01 Hz, 0.1 Hz, 0.2 Hz, 0.5 Hz, and 1 Hz were applied to the MR damper using an MTS electro-hydraulic servo testing system. The excitation current was regulated in real time according to the measured displacement and velocity of the MR damper, thereby maintaining the desired magnetic field intensity within the damping channel. Under these operating conditions, a series of representative damping force–displacement response curves were obtained. The experimental results demonstrate that the LS-DJAM accurately characterizes the dynamic hysteresis behavior of the MR damper across different excitation frequencies, verifying its robustness and providing a solid basis for further model refinement and engineering applications.

**Figure 11:**
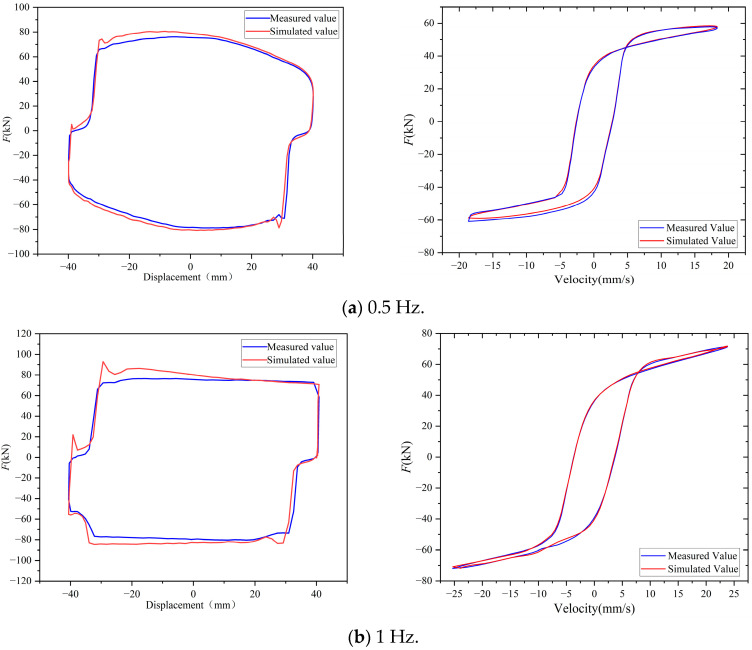
Comparison of output damping force–displacement and force–velocity curves of magnetorheological damper under different working conditions.

In this section, to comprehensively assess LS-DJAM’s capability in modeling rate-dependent dynamic hysteresis, sinusoidal excitation signals at multiple frequencies were applied to the MR damper mechanical model. The resulting force–displacement output curves are depicted in Figure 11. Table 4 presents the error metrics for displacements from −40 mm to 40 mm at different frequencies shown in Figure 11, including peak-to-valley (PV), average (AVG), and standard deviation (STD) values. The peak-to-valley value represents the difference between the maximum and minimum errors, with a smaller PV indicating lower random error in the model. The average reflects the central tendency of the errors; an average closer to zero indicates higher modeling accuracy. The standard deviation quantifies the dispersion of errors around the mean; a smaller standard deviation indicates reduced error fluctuation. The experimental results demonstrate that the proposed mathematical model exhibits excellent accuracy and robustness. Data analysis indicates minor fluctuations in LS-DJAM’s accuracy during modeling. Although these fluctuations are within acceptable limits, their underlying causes merit further investigation.

The force–velocity hysteretic behaviors of the MR damper are compared under 0.5 Hz and 1 Hz conditions, where the simulation curves show exceptional consistency with the experimental data throughout the velocity range. The LS-DJAM model precisely replicates the yield stress-dominated nonlinear transitions at 0.5 Hz while effectively capturing the dynamic hysteresis loop expansion resulting from enhanced eddy current and excess losses at 1 Hz. Such high-fidelity fitting across frequency domains robustly substantiates the validity of the proposed loss separation theory and the excellent global search capability of the PSO–GA. Consequently, the proposed model significantly mitigates the limitations of conventional static models, offering a rigorous theoretical framework for the accurate characterization of the damper’s dynamic response.

### 4.3. Performance Comparison with Other Models

**Figure 12:**
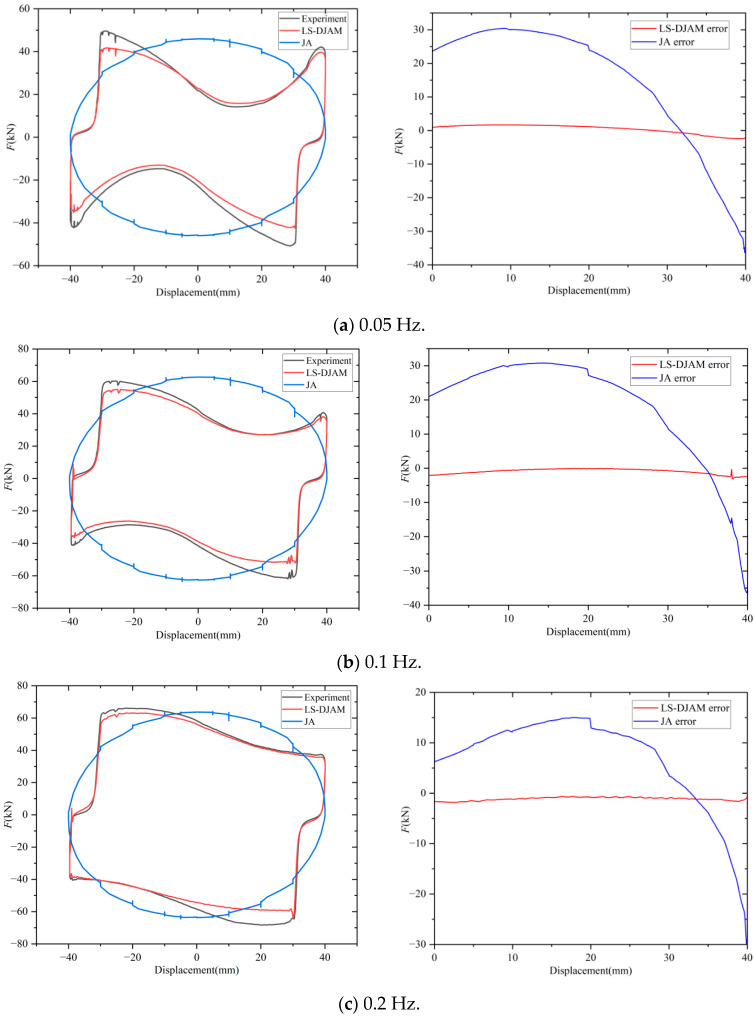
Comparison of simulation results and error maps of JA model and LS-DJAM of MRD under different working conditions.

To further validate LS-DJAM’s accuracy in representing MR damper hysteresis characteristics, a static JA hysteresis model was simulated in MATLAB/Simulink (2024a) for comparison with LS-DJAM. The force–displacement output curves and corresponding errors for displacements from 0 to 40 mm for both models are presented in Figure 12.

Comparative analysis under three vibration frequencies shows that at 0.05 Hz, the maximum deviation between the JA model simulation and the measured damping force reaches 31.4 kN at a damper displacement of 8 mm. In contrast, the LS-DJAM model exhibits a maximum error of 8.85 kN at the same displacement, with a root mean square error (RMSE) of 3.922 kN, corresponding to an 87.5% reduction in the maximum deviation.

**Figure 13:**
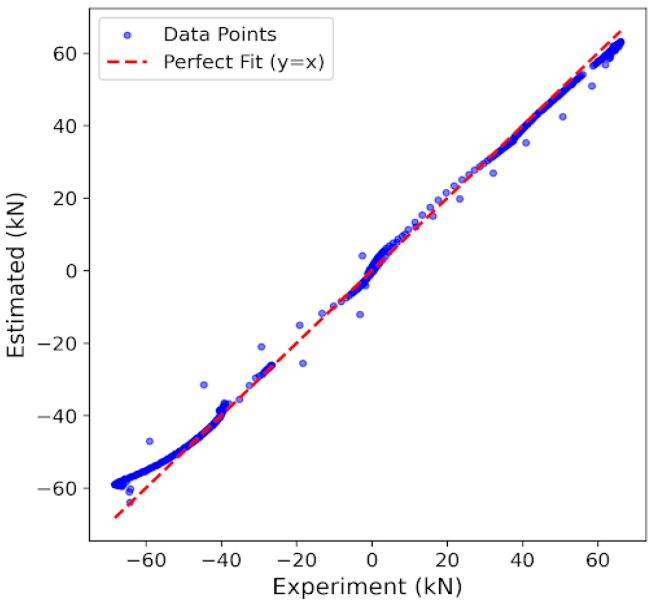
Regression analysis of the actual and predicted forces under the 0.2 Hz operating condition.

At 0.1 Hz, the JA model shows a maximum error of 27.6 kN at 11 mm displacement, while the LS-DJAM model achieves a maximum error of 12.11 kN and RMSE of 3.85 kN, yielding a 56.1% reduction in maximum error. At 0.2 Hz, the JA model’s maximum error reaches 14.9 kN at 14 mm displacement, whereas LS-DJAM demonstrates a maximum deviation of 13.15 kN and RMSE of 3.52 kN, corresponding to an 11.7% reduction in the maximum error. Figure 13 provides a further regression analysis between the measured and predicted forces at 0.2 Hz. As depicted in the scatter plot, where the abscissa and ordinate denote the experimental and estimated forces respectively, the data points are densely concentrated along the y = x diagonal. This pattern suggests an exceptionally strong linear correlation between the model outputs and the experimental data. This consistency is quantitatively corroborated by the statistical indicators, with an R^2^ of 0.9942. These findings confirm that the identified model is capable of accurately capturing the complex nonlinear hysteretic behavior of the MR damper with negligible error. These results indicate that LS-DJAM significantly improves the modeling performance over the static JA model.

In contrast, the LS-DJAM, which incorporates energy dissipation mechanisms, agrees well with the experimental results. This discrepancy arises because the magnetic particle chain structures in the MR fluid readily rupture and reorganize under weak magnetic fields, leading to pronounced dissipative and relaxation phenomena. As a result, the real damping force decays rapidly toward zero near the origin, producing a contracted hysteresis loop in the experimental data. By introducing a loss-related term into the JA framework, LS-DJAM effectively captures chain yielding, breakup, and viscous flow under low-field and small-strain conditions. Consequently, the LS-DJAM accurately reproduces the rapid relaxation behavior and the corresponding shrinkage of the force–displacement curve near zero displacement, rather than exhibiting the unrealistic peak predicted by the JA model. These results indicate that LS-DJAM significantly improves the modeling performance over the static JA model. Compared to common dynamic models, the proposed LS-DJAM demonstrates significant advantages. The dynamic Bouc–Wen model, while effective for nonlinear fitting, is fundamentally phenomenological; its parameters lack physical interpretation, limiting its utility for material or design optimization [43]. Similarly, empirical dynamic JA models rely on data-driven coefficients, often leading to poor generalization under varying frequencies due to the absence of micro-physical descriptions. Conversely, the LS-DJAM preserves the physical integrity by incorporating Bertotti’s statistical loss theory. By resolving losses through physically meaningful parameters, it not only outperforms the static JA model in accuracy but also elucidates dynamic energy dissipation mechanisms, striking a balance between physical insight and modeling precision. Although validated on the MRD-SEU-D050, the specific parameter values are not directly transferable to other designs, as Jiles–Atherton parameters (*M_s_*, *k*) are intrinsic to material properties and magnetic circuit geometry. Consequently, parameter re-identification is necessary when hardware changes. However, the proposed modeling framework and identification strategy exhibit high scalability. Since the underlying loss separation theory is universal, the LS-DJAM structure remains valid for various MR dampers regardless of size or shape. Moreover, the developed PSO–GA provides a robust, automated tool for parameter re-identification, eliminating the need for manual calibration and ensuring the method’s practical engineering versatility.

In this study, LS-DJAM is coupled with the Bingham mechanical model, and the PSO–GA is used to identify model parameters. The results show that the deviation between simulated damping forces and experimental values is substantially reduced and within acceptable limits, although not yet fully optimized. Detailed analysis suggests two main reasons: first, the MR damper’s internal structure is complex, with excitation coils, pistons, and cylinders all contributing to hysteresis. LS-DJAM does not consider their coupling effects, potentially limiting the output force accuracy. Second, the Bingham model cannot fully capture the MR damper’s complex mechanical behavior. Although simple and convenient, it cannot accurately represent the MRF stress–strain process, particularly shear-thinning behavior. Moreover, MRF rheology is sensitive to temperature and particle sedimentation, so environmental variations can alter its yield stress.

## 5. Conclusions

This study develops a dynamic JA model based on loss statistics (LS-DJAM) to capture MR damper hysteresis, with key parameters identified via a particle swarm–genetic algorithm (PSO–GA). Its effectiveness is verified against experimental data under multiple operating conditions.

(a) LS-DJAM captures the nonlinear *B–I* relationship in the MR damper, incorporating eddy current and residual losses as well as frequency-dependent hysteresis. This enables the accurate prediction of the MR damper output force, supporting numerical analysis and engineering applications.

(b) PSO–GA integrates genetic operators into PSO, overcoming premature convergence and slow optimization while improving parameter identification accuracy. Key JA parameters identified via PSO–GA were combined with the Bingham model in Simulink, producing damper outputs closely matching the experimental data and reducing errors, confirming the practical applicability.

Despite its strong performance, the LS-DJAM–Bingham model can be further improved. The MR damper’s internal components (oils, pistons, and cylinders) generate coupled hysteresis which is not fully captured. Further refinement and real-time modeling methods are needed to optimize the structure and enhance the predictive capability for practical rapid-response applications.

## Figures and Tables

**Table 1:** Hyperparameter settings of the proposed PSO–GA.

PSO Strategy			GA Strategy			
Inertia Weight	Learning Factors	Velocity Limit	Selection Strategy	Crossover Probability	Mutation Probability	Convergence Criteria
0.2–0.8	2	[−0.5, 0.5]	Roulette Wheel Selection	0.8	0.1	Max Iterations

**Table 2:** Parameter identification results.

Parameter	*M_s_* (A/m)	*α*	*a* (A/m)	*c*	*k* (A/m)
Improved PSO	1.31 × 10^6^	8.92 × 10^−5^	25.32	0.19	66.51
PSO	1.27 × 10^6^	7.61 × 10^−5^	20.03	0.25	70.96
PSO–GA	1.31 × 10^6^	8.40 × 10^−5^	25.32	0.20	66.42
Theoretical Value	1.32 × 10^6^	8.43 × 10^−5^	25.32	0.20	66.61

**Table 3:** MR fluid parameters.

Granule Diameter (μm)	Cladding Thickness (μm)	Inter-Particle Spacing (μm)	Volume Fraction (%)	Zero-Field Viscosity (Pa·s)
1.5	0.015	0.5	35	2

**Table 4:** Prediction error.

	PV (kN)	AVG (kN)	STD (kN)
0.5 Hz	8.5	3.5	4.3
1 Hz	16.1	3.6	4.4

## Data Availability

Data are contained within the article.
